# Modification of wheat gluten for improvement of binding capacity with keratin in hair

**DOI:** 10.1098/rsos.171216

**Published:** 2018-02-07

**Authors:** Shukun Wang, Danyang Meng, Sisi Wang, Zhong Zhang, Ruijin Yang, Wei Zhao

**Affiliations:** 1 State Key Laboratory of Food Science and Technology, Jiangnan University, No. 1800 Lihu Road, Wuxi 214122, People's Republic of China; 2 School of Food Science and Technology, Jiangnan University, No. 1800 Lihu Road, Wuxi 214122, People's Republic of China; 3 Department of Food Science and Technology, University of Nebraska–Lincoln, Food Innovation Center, 1901 N 21st Street, Lincoln, NE 68588-6205, USA

**Keywords:** wheat gluten, hydrolysis, quaternization, keratin

## Abstract

In this study, enzymatic hydrolysis and cationization with epoxypropyldodecyldimethylammonium chloride of wheat protein, an economic protein complex containing great amount of disulfide bonds, were conducted to improve properties such as solubility and disassociation behaviour for recovery of damaged hair when used in shampoo. The optimal conditions for enzymatic hydrolysis were pH 8.2, 55°C with Alcalase for 60 min. After the selected hydrolysis, the degree of hydrolysis, nitrogen solubility index, foaming capacity index, foam stability index, emulsifying activity index and emulsion stability index of hydrolysate with 58.71% of short-chain peptides (less than 1000 Da) were 8.81%, 39.07%, 225%, 56.67%, 9.62 m^2^ g^−1^ and 49.08, respectively. The cationization was followed to raise the isoelectric point of wheat protein hydrolysate from 7.0 to 10.0, which could facilitate the quaternized protein hydrolysate to adhere to the surface of hair at the range of pH 5–6 of hair care products to form more disulfide bonds. The results show that a shampoo with quaternized wheat proteins hydrolysate possesses excellent properties in recovering damaged hair, making the surface of hair smooth and compact.

## 1. Introduction

Wheat protein, by-product of wheat starch, is derived from wheat flour by separation, extraction and drying. It is available in large amounts at a relatively low price [1,2]. With the boost of safety awareness, healthy ingredients like natural proteins are undoubtedly needed in hair care products. Studies have shown that human hair is a filamentous biomaterial and 90% (by weight) of it is keratin [3], which is characterized by high concentration of disulfide bonds. Hair damage results from both mechanical and chemical trauma, such as sunlight, bleaches, straighteners and hair dyes, altering the physical structural components of hair [4]. The damage of hair leads to the oxidative cleavage of disulfide bonds [5]. The main driving forces to strengthen human hair are the hydrophobic interactions and disulfide bonds between small peptides and human hair keratins [6]. Therefore, the wheat proteins composed by gliadin and glutenin [7], with abundant disulfide bonds are promising to be an effective and healthy ingredient to recover damaged hair by connecting the free sulfydryl exposed on the hair surface and the free sulfydryl exposed from the inner structure of protein through enzymatic hydrolysis.

For the past decades, lots of methods including physical, chemical and enzymatic methods have been used to modify wheat gluten [8–11], and many types of protein hydrolysate from plants and animals have been used in hair repair products [6,12]. However, few were focused on the disassociation behaviour of protein hydrolysate in hair care products which is not suitable for adherence. Keratin whose isoelectric point is 3.8 [13] is negatively charged when applied with hair care product whose pH value is 5–6, whereas most protein hydrolysate is neutral under that condition, which makes adherence difficult. Therefore, finding a way to change the isoelectric point of protein hydrolysate is of great importance.

In this paper, enzymatic hydrolysis and cationization with epoxypropyldodecyldimethylammonium chloride (EDDAC) of wheat proteins were conducted to improve properties such as solubility and binding capacity with keratin for better application in hair care products. A shampoo with quaternized wheat proteins hydrolysate possessed excellent properties in recovering damaged hair, making the surface of hair smooth and compact.

## 2. Material and methods

### 2.1. Materials

Vital wheat gluten with 71.48 ± 1.32% (w/w, dry basis) crude protein content (*N* × 5.7) [14,15] and 7.90 ± 0.11% moisture was obtained from Jiangsu Lianhai Biological Technology Co. Ltd (Jiangsu, China). Alcalase (activity 2.4 U g^−1^, optimum pH 8.2 and temperature 55°C), Neutralprotease (activity 1.5 AU g^−1^, optimum pH 7.0 and temperature 50°C) and Flavorzyme (activity 500 LAPU g^−1^, optimum pH 6.8 and temperature 50°C) were purchased from Novozyme (Shanghai, China). Folin–Ciocalteu was purchased from Sigma. Soya bean oil was obtained from Jinlongyu Co. Ltd *N*,*N*-dimethyldodecylamine (DDA) was purchased from TCI (Shanghai) Development Co. Ltd (Shanghai, China). All other chemicals were of analytical grade except those used in chromatography.

### 2.2. Hydrolysis reaction

The hydrolysis was carried out using a 1 l double-walled stainless steel vessel connected to a water bath. Each experiment was conducted using a total mass of 600 g reaction mixture in which solid concentration was 10% (w/w) and enzyme–substrate ratio was 150 U g^−1^. Hydrolysis reactions were conducted under stable pH and temperature conditions, that is, pH 8.2, 55°C for Alcalase, pH 7, 50°C for Neutralprotease, pH 6.8, 50°C for Flavorzyme. The pH was adjusted to suitable level by 0.1 M NaOH.

After the addition of enzyme liquor to substrate solution, the double-walled glass vessel was closed with a clasp to avoid evaporation of water and stirred using an overhead stirrer at 40 r.p.m. The enzyme in the mixture was inactivated by heating at 100°C for 10 min in a water bath after reaction. The samples were then spray dried immediately. The spray-dried samples were stored for further analysis.

### 2.3. Determination of the degree of hydrolysis

The degree of hydrolysis (DH) is defined as the percentage of cleaved peptide bonds to the total number of bonds per unit weight. The DH was measured by the pH-Stat method as described previously with minor modifications. The equation is as follows:

$$\text{DH}=\frac{100\times B\times N_{b}}{\alpha \times m_{p}\times h_{\mathrm{tot}}}\times 100\%,$$

where *B* is the volume of NaOH solution consumed during the reaction (ml), *N_b_* is the concentration of NaOH solution used during the reaction (mol l^−1^), *α* is the average dissociation degree of α-NH_2_ in wheat gluten, *m_p_* is the weight of wheat gluten, *h*_tot_ is the total number of peptide bonds in protein substrate. *h*_tot_ = 8.3 and *α *= 1 [16].

### 2.4. Measurement of viscosity

The viscosity of the sample taken from the reaction vessel was determined using a rotational viscometer (NDJ-5S, Shanghai Jingtian Electronic Instrument Co. Ltd, Shanghai, China), immediately. Each sample was repeatedly measured three times and the average value was taken.

### 2.5. Measurement of nitrogen solubility index

Wheat gluten and spray-dried samples were dissolved in deionized water, formulated into 1% (w/v) solution and agitated at ambient temperature for 1 h. After centrifugation at 4000 r.p.m. for 20 min at 4°C, the supernatant was taken and the protein content was measured by reference to Barnes [17].

Nitrogen solubility index (NSI) = protein content in the supernatant/total protein content in the sample × 100%.

### 2.6. Measurement of foaming property

The proteins were dissolved in deionized water to prepare a 1% (w/v) solution, 30 ml of which was taken and whipped at 10 000 r.p.m. for 1 min. The foam volume *V*_1_ was recorded immediately. *V*_2_ was recorded 10 min later. Foaming capacity index was calculated as *V*_1_/30 × 100%. Foam stability index was calculated as *V*_1_/*V*_2_.

### 2.7. Determination of emulsifying property

The emulsifying property was determined by the method of Pearce & Kinsella [18] with minor modifications. To prepare the emulsions, 5 ml of soya bean oil and 35 ml of 1% (w/v) samples were shaken together and homogenized in a blender at 10 000 r.p.m. for 1 min at room temperature. Then samples were taken from the bottom of container at different times (0 and 10 min) and diluted with 0.1% sodium dodecylsulfate (SDS) (w/v) by 100 times. The absorbance was measured at 500 nm wavelength with 0.1% SDS (w/v) as a blank. Emulsifying activity index (EAI) was calculated as follows:

$$\text{EAI}=\frac{2\times 2.303A\times N\times 10^{-4}}{\varphi LC},$$

where *N* is the dilution ratio, *φ* is the fraction of oil in the system, *L* is the light path of the colorimetric cell (1 cm), *C* is the concentration of protein. Emulsion stability index (ESI) is calculated as follows:

$$\text{ESI}=\frac{A_{0}\times \Delta T}{\left(A_{0}-A_{t}\right)},$$

where *ΔT* is 10, *A*_0_ is the absorbance at 0 time, *A_t_* is the absorbance at *t* time.

### 2.8. Molecular weight distribution

Molecular weight distribution profiles of the deamidated wheat gluten hydrolysate were determined by size-exclusion HPLC (Agilent HP1100, USA) with a TSKgel 2000 SWXL (300 mm × 7.8 mm) column. The temperature was 30°C. The mobile phase consisted of acetonitrile (40), water (60) and trifluoroacetic acid (0.1). The flow rate of the mobile phase was 0.5 ml min^−1^. The absorbance was monitored with UV detection at 220 nm.

A solution of 100 mg of sample diluted to 10 ml in the mobile phase was treated ultrasonically for 5 min to dissolve and centrifuged (Velocity 14R, Dynamic, Austria) at 10 000 r.p.m. After centrifugation and microfiltration, the sample was injected to be analysed. A mixture of four-protein standards containing cytochrome C (12 384 Da), bacillus enzyme (1422 Da), acetic acid–acetic acid–tyrosine–arginine (451 Da), acetic acid–acetic acid–acetic acid (189 Da) was taken to calibrate the column and make the standard curve. The plot of logMW against elution time was constructed, and the molecular weight distribution for each hydrolysate was then calculated according to the plot. Waters GPC software was used to analyse the chromatographic data.

### 2.9. The synthesis of epoxypropyldodecyldimethylammonium chloride

DDA was added to 1.5 M hydrochloric acid dropwise under magnetic stirring and the final ratio was 1 : 1. The solution was slowly heated to 50°C, and then epichlorohydrin (ECH) was added dropwise. The molar ratio of DDA to ECH was 0.95 : 1. When the pH of the mixture no longer changed, stirring was stopped and the system cooled to room temperature, then the reaction was ended. NaOH solution of 25% (w/v) was added to adjust pH value to 11. The product was vacuum distilled and washed with anhydrous ether to remove the solvent. After three times of repeated centrifugation, the sample was vacuum dried at 20°C for 24 h. White or light yellow solid was obtained and stored for subsequent use.

### 2.10. Quaternization of wheat protein hydrolysate

The wheat protein hydrolysate (WPH) was added into the EDDAC liquor with 0.8 : 1 mole ratio of α-NH_2_ to EDDAC. The mixture was stirred and heated at 50°C for 2 h under stable pH value. Lactic acid was added dropwise into the reaction system until the pH value of the liquid was 5. The reaction mixture was condensed and purified with 95% ethyl alcohol to remove organic impurities and NaCl. The quaternized wheat protein hydrolysate was obtained after filtration and vacuum drying.

### 2.11. Measurement of the content of disulfide bonds, free sulfydryl and total sulfydryl

The content of disulfide bond, free sulfydryl and total sulfydryl were measured by the method of Beveridge *et al*. [13]. Sulfydryl can react with dithiobisnitrobenzoic acid producing sulfo-nitrobenzene ion at pH 8.0 whose absorbance at 412 nm is proportional to content. The total sulfydryl was measured after the disulfide bonds in protein were reduced by 2-mercaptoethanol into sulfydryl. The content of disulfide bonds was calculated as follows.

The content of disulfide bonds = (the content of total sulfydryl – the content of free sulfydryl)/2.

### 2.12. Measurement of the content of α-NH_2_

The pH value of WPH or reactant solution was adjusted to be 8.2 by HCl solution of 0.1 M in a flask. The mixture was slowly added with 10 ml of formaldehyde solution, and then titrated with NaOH solution (0.1 M) until the pH value was 9.2. The content of α-NH_2_ was calculated as follows:

$$B=\frac{C_{1}(V_{1}-V_{0})}{m},$$

where *B* is the content of α-NH_2_ (mmol g^−1^), *m* is the mass of sample (g), *C*_1_ is the concentration of NaOH solution (mol l^−1^), *V*_1_ is the volume of NaOH solution consumed in titration (ml), *V*_0_ is the volume of NaOH solution consumed in blank test (ml).

### 2.13. Formula of conditioning shampoo

The formula of conditioning shampoo is shown as below.

**Table:** 

components	mass per cent
citric acid	0.05%–0.1%
fatty alcohol polyoxyethylene ether sodium sulfate (SES)	20%
cocamidopropyl betaine	3%
sulfated coconut oil fatty acid monoethanolamide sodium	1%
octadecanol	0.8%
pearling agent	1.5%
casson	0.05%
essence	0.8%
water	72.75%–72.8%

### 2.14. Characterization

A Fourier transform infrared (FTIR) spectrum was recorded with a NEXUS 670 IR spectrometer (Nicolet, USA) between 400 and 4000 cm^−1^. Sample of 1–2 mg was thoroughly mixed with 200 mg KBr and ground before assay. Both the sample and KBr were dehydrated and ground to less than 2 µm to eliminate the influence of scattered light.

Scanning electron microscopy (SEM) images were obtained with a Hitachi TM3030 field emission scanning electron microscope. Hair fibres were taken randomly from the hair tress and mounted onto aluminium stubs using conductive carbon adhesive tape, and sputter coated from a gold leaf source to impart conductivity to the surface of sample for 30 s. The microscope was operated at 10 kV, and samples were viewed at 1500× magnification.

### 2.15. Statistical analyses

Each experiment was carried out at least in triplicate. A *p* value of 0.05 was used to determine statistical significance in all tests.

## 3. Results and discussion

### 3.1. Enzymatic hydrolysis of wheat gluten

**Figure 1.**
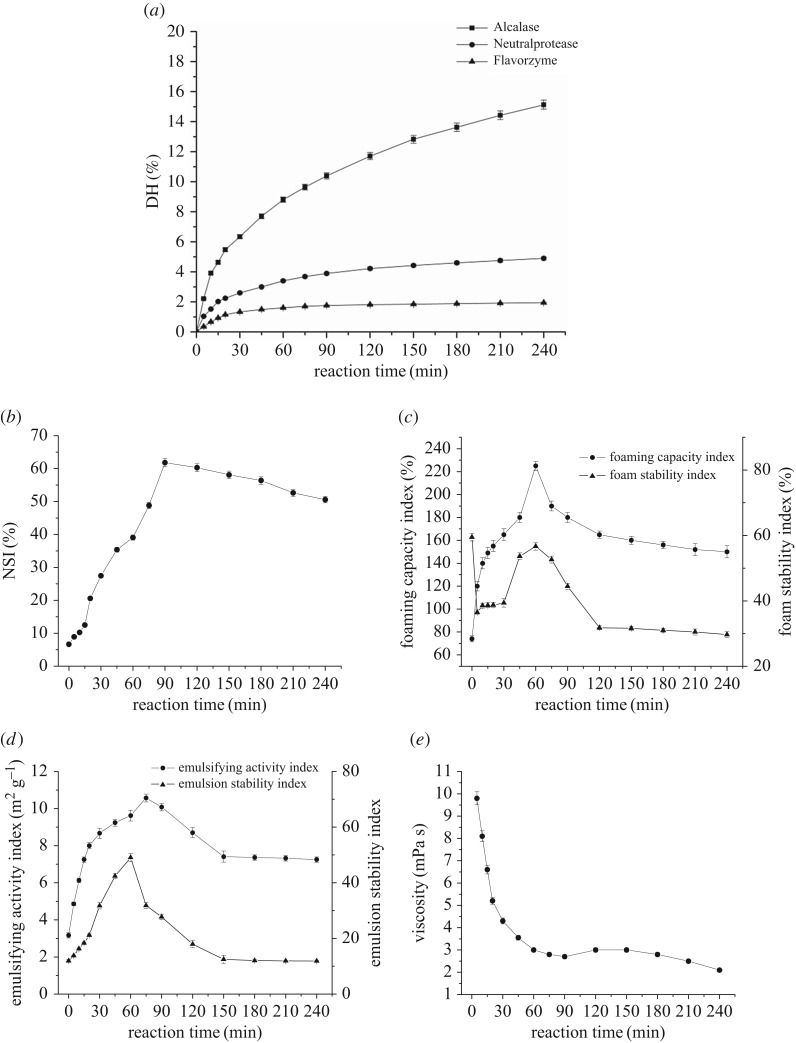
Enzymatic hydrolysis curves of wheat gluten with different protease (*a*). Changing curves of NSI (*b*), foaming properties (*c*), emulsifying properties (*d*) and viscosity (*e*) with Alcalase.

The hydrolysis conditions including enzyme (Alcalase, Neutral protease and Flavorzyme with 150 U g^−1^ enzyme–substrate ratio) and reaction time were optimized according to the DH, properties of hydrolysate, molecular weight distribution of hydrolysate and the content of free sulfydryl. The hydrolyses of wheat gluten with different enzyme (Alcalase, Neutral protease and Flavorzyme) all proceeded at a rapid rate during the first 20 min and slowed down thereafter (figure 1*a*), which is a typical hydrolysis curve for protein [19]. The DH values at 240 min were 15.13%, 4.90% and 1.94%, respectively, showing that the Alcalase catalysed the degradation most efficiently, which is consistent with the results of Adamson & Reynolds [20]. Thus, only Alcalase was used for further research.

The properties of hydrolysate including viscosity, NSI, foaming properties and emulsifying properties were measured during the reaction (figure 1*b,c,d*). As the reaction proceeded, the wheat protein was hydrolysed to be free amino acid and short-chain peptides leading to the sharp decline of viscosity. The viscosity levelled off with a slight increase at 90 min (figure 1*e*). The slight increase of viscosity can be explained by plastein reaction, a reverse reaction of hydrolysis forming a protein-like substrate under certain conditions causing the rebound of viscosity [21]. Other indexes of properties (NSI, foaming capacity index, foam stability index, EAI and ESI) exhibited a similar tendency that increased firstly and then decreased at time ranging from 60 to 90 min (figure 1*c,d,e*). The peak value was 61.8%, 225%, 56.67%, 10.57 m^2^ g^−1^ and 49.08, respectively. Considering all of these indicators, the proper hydrolysis time can be chosen from 60 to 90 min.

**Figure 2.**
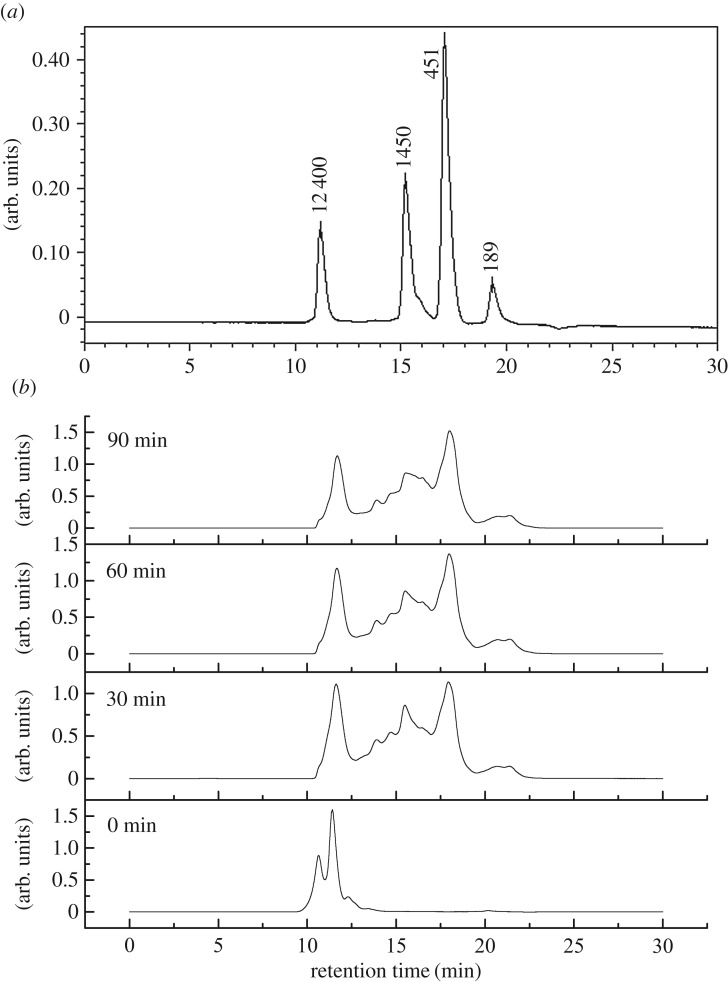
The standard curve of molecular weight (*a*) and phase diagram of polypeptide distribution of wheat gluten and hydrolysate (*b*).

Figure 2 shows the chromatogram profiles of the original wheat gluten and WPH obtained by the treatment of Alcalase for different times. Compared with the original wheat gluten, the peaks of WPH moved to the direction of long retention time as the DH increased. After 30 min of enzymatic hydrolysis, the peak at 11 min disappeared and several new peaks appeared at elution time ranging from 13 to 20 min. The appearance of new peaks is in agreement with what Wang *et al*. reported [22], but the hydrolysis time and the number of new peaks are different because different kinds of enzyme were used. The results demonstrated that the hydrolysis of wheat protein produced some substances with lower relative molecular weight. The molecular weight distribution of wheat gluten and hydrolysate shown in table 1 made further evidence. The polypeptides with molecular weight more than 10 000 Da were totally degraded in 30 min from 81.82% to 0% and the polypeptides with molecular weight between 180 and 3000 Da were produced. As the hydrolysis proceeded for 60 min, the molecular weight of more than 50% of peptides is less than 1000 Da, which did not change significantly for the next 30 min.

**Table 1.** Molecular weight distribution of wheat gluten hydrolysates.

	percentage			
relative molecular weight	0 min	30 min	60 min	90 min
<180	0	8.95	11.19	11.42
180–500	0	28.37	30.24	32.24
500–1000	0	17.20	17.28	18.06
1000–2000	0	13.60	12.35	11.77
2000–3000	0	5.78	5.05	4.65
3000–5000	2.33	4.51	4.09	3.85
5000–10 000	15.85	21.59	19.81	18.00
>10 000	81.82	0	0	0

**Figure 3.**
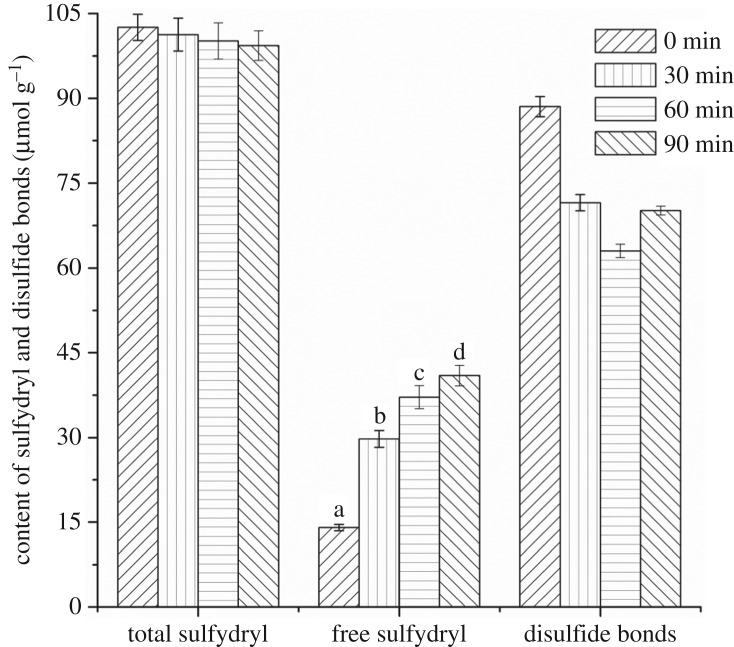
Content of disulfide bonds, free sulfydryl and total sulfydryl.

It is shown in figure 3 that with the process of hydrolysis from 0 to 90 min, the amount of total sulfydryl did not change apparently, but the content of free sulfydryl increased from 14.03 to 40.96 µmol g^−1^ and the content of disulfide bonds decreased from 44.26 to 29.18 µmol g^−1^. Since disulfide bonds cannot be cleavaged through hydrolysis, a possible explanation is that the free sulfydryl in the inner structure of wheat gluten which could not be measured was exposed. Since the amount of disulfide bonds were calculated through the content of total sulfydryl and free sulfydryl, with the measured content of free sulfydryl increasing, the amount of disulfide bonds appeared to decrease.

All related aspects considered, the conditions of the hydrolysis were optimized to be pH 8.2, 55°C with Alcalase for 60 min. The DH, NSI, foaming capacity index, foam stability index, EAI and ESI of hydrolysate were 8.81%, 39.07%, 225%, 56.67%, 9.62 m^2^/g and 49.08, respectively. The peptides with molecular weight less than 1000 Da accounted for 58.71%.

### 3.2. Quaternization of wheat protein hydrolysate

**Figure 4.**
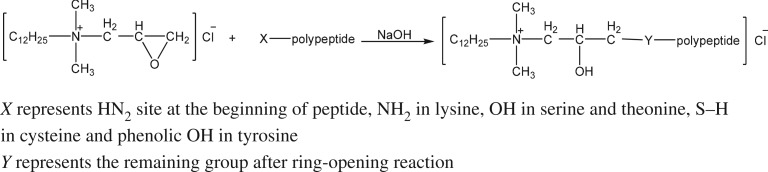
The chemical reaction equation of quaternized wheat protein hydrolysate.

The quaternization of WPH is the cycloaddition reaction between α-NH_2_ which is in the protein hydrolysate and the epoxy bond in quaternary ammonium salt (figure 4). Besides the HN_2_ at the beginning of peptide, there are many other sites such as NH_2_ in lysine, OH in serine and theonine that can react with EDDAC. But the content of these reaction sites is small compared to the content of HN_2_ site at the beginning of peptide. Therefore, the majority of the reaction took place at the HN_2_ site at the beginning of peptide.

**Figure 5.**
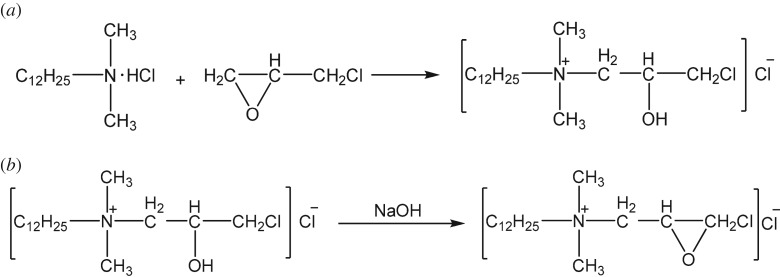
Chemical reaction equation of synthesis of EDDAC. (*a*) Cycloaddition reaction. (*b*) Ring-closing reaction.

**Figure 6.**
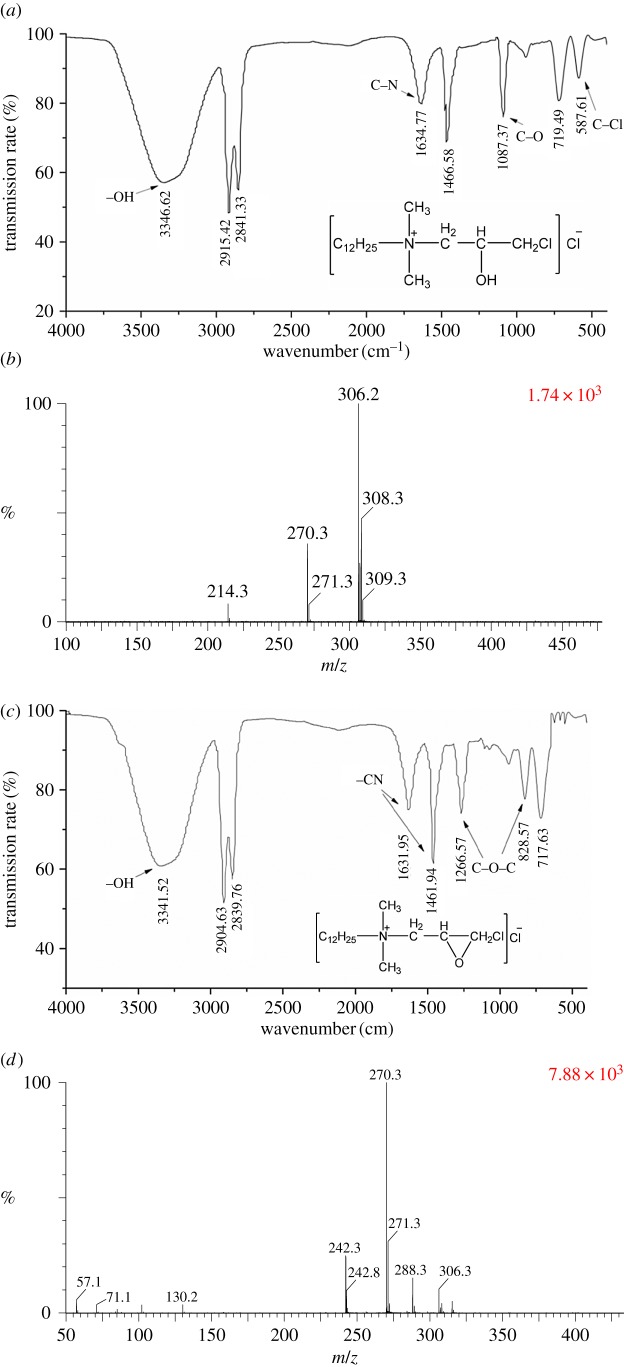
Infrared spectrum (*a*) and mass spectrum (*b*) of the first step product of synthesis of reaction of EPDDAC. Infrared spectrum (*c*) and mass spectrum (*d*) of the second step product of synthesis of reaction of EPDDAC.

It took two steps to synthesize EDDAC: the cycloaddition reaction (figure 5*a*) [23,24] and the ring-closure reaction (figure 5*b*). The reaction intermediate 3-chloro-2-hydroxypropyldimethyldodecylammonium chloride (CHDDAC) was produced with 0.95 : 1 mole ratio of DDA to ECH at 50°C for 180 min. Figure 6*a* shows the FTIR spectrum of CHDDAC. The wide band at 3346.62 cm^−1^ was characteristic of associate –OH groups. The peaks at 2841.33 and 2915.42 cm^−1^ corresponded with stretching vibration of methyl and methylene in hydrocarbon. The band at 1634.77 cm^−1^ represented the bending vibration of C–N. The peak at 1466.58 cm^−1^ was the characteristic absorption of C–N in quaternary ammonium salt. The peak at 1087.37 cm^−1^ was the stretching vibration of C–O in C–OH. The spectral band at 719.49 cm^−1^ meant swaying in-plane vibration of CH_2_. The peak at 587 cm^−1^ corresponded with the C–Cl stretching vibration. All these structural features were in line with the target product, CHDDAC.

The sample was then bombarded with an electrospray ionization source, and the mass spectrum of the sample was obtained, as shown in figure 6*b*. The ion peak at 306 showed that the sample contained chlorine atoms, because 306 plus 35.5 (relative molecular weight of Cl^−^) is 341.5, which is in accordance with the relative molecular weight of CHDDAC. The ion peak at 214 was the long-chain tertiary amine quaternary ammonium group, which may be the fragment ion peak or the ion peak of the residual tertiary amine in the reaction system. The peaks at 270.3 and 271.3 were the positive ions of the EDDAC, which was in line with the product molecular structure and reaction characteristics.

The FTIR spectrum of the product is shown in figure 6*c*. The wide band at 3341.52 cm^−1^ was characteristic of –OH groups. The peaks at 2839.76 and 2904.63 cm^−1^ corresponded with hydrocarbon stretching vibration in methyl and methylene. The band at 1631.95 cm^−1^ was the characteristic absorption of C–N. The peak at 1461.94 cm^−1^ was assigned to the vibration of C–N in quaternary ammonium salt. The peaks at 1266.57 and 828.57 cm^−1^ corresponded with epoxy propyl stretching vibration in the ring C–O–C. The peak at 717.53 cm^−1^ was the in-plane vibration swing of CH_2_. No present C–Cl absorption peak, contrasting to figure 6*a*, conformed to the structure characteristics of the target product. The above results show that the C–OH has been closed with –CH_2_Cl and the target product was generated.

Figure 6*d* is the mass spectrum of the product. The ion peaks at 57.1 and 71.1 represent the carbon chains CH_3_CH_2_CH_2_CH_2_– and CH_3_CH_2_CH_2_CH_2_CH_2_–, respectively. The ion peak at 270 was the signal of the cationic part of EDDAC, because 270 plus 35.5 (relative molecular weight of Cl^−^) is 341.5, which is in accordance with the relative molecular weight of EDDAC. All these information demonstrated that EDDAC was synthesized.

**Figure 7.**
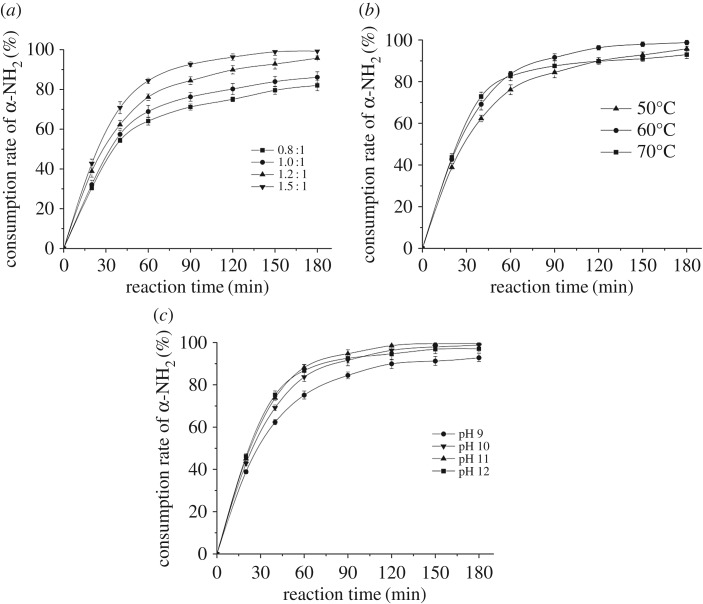
Influence of mole ratio (*a*), temperature (*b*) and pH (*c*) on reaction of cationizing the wheat protein hydrolysate.

The quaternization reaction was optimized through three factors: mole ratio of EDDAC to WPH, temperature and pH value. Figure 7*a* shows that with the extension of reaction time, the α-NH_2_ was constantly consumed. Consumption rates of α-NH_2_ reached 82.02%, 86.15%, 95.76% and 86.15% at 180 min under the mole ratio of 0.8 : 1, 1 : 1, 1.2 : 1 and 1.5 : 1 respectively, showing the increase of the EDDAC was in favour of the quaternization. Theoretically, any kind of increase of reactant will be positive to promote the reaction, but in practice, when the amount of α-NH_2_ was more than EDDAC, the surplus protein will make the system too thick to convey mass and heat, thus made the reaction slow down. As the mole ratio increased, sufficient EDDAC increased the reaction velocity gradually, but as reaction proceeded, all of the reaction velocity slowed down indicating the end of the reaction. From the view of economy, the mole ratio between EDDAC and WPH was made 1.2 : 1.

In figure 7*b*, in the early stage of the reaction, the higher the temperature was, the higher the α-NH_2_ consumption rate was. The optimal consumption rate appeared at the temperature of 60°C. Higher temperature would provide more energy to reach the activation energy of the reaction and accelerate the process, but when the temperature was too high, a variety of side reactions frequently occur because of superfluous energy, which is not conducive to the right direction of the reaction. Therefore, 60°C was the most appropriate reaction temperature.

Within the pH scope of 9–11, the consumption rate of α-NH_2_ increased with the increase of pH value (figure 7*c*). The reaction system of pH 12 proceeded quickly in the early stage, but was slower than that of pH 11 later. It can be explained that the product was easy to be hydrolysed when pH value was 12. In the reaction system of pH 11, the consumption of α-NH_2_ was 94.76%, 98.55%, 98.55% and 99.48% at 90, 120, 150, 180 min, respectively. Therefore, the pH value of reaction was set to be 11 and the reaction time was 120 min.

**Figure 8.**
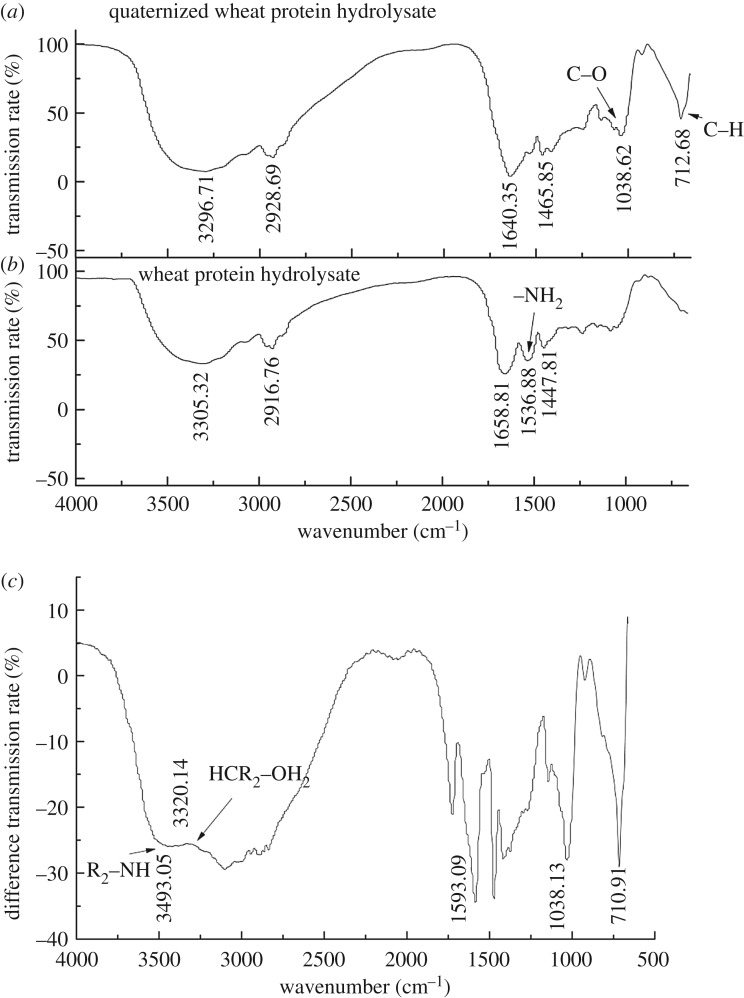
The infrared spectra of quaternized wheat protein hydrolysate (*a*), wheat protein hydrolysate (*b*) and (*c*) when transmission rate of wheat protein hydrolysate was subtracted from that of quaternized wheat hydrolysate.

The quaternization reaction was performed under optimum conditions. Compared with WPH, the FTIR spectrum of quaternized wheat protein hydrolysate (QWPH) shows obvious changes (figure 8). The peak at 1536.88 cm^−1^ which is the characteristic absorption peak for the amino disappeared in the FTIR spectrum of QWPH. At the same time, the peaks at 1038.62 and 712.68 cm^−1^ which corresponded with the C–O stretching vibration in C–OH and C–H in-plane vibration swing of –CH_2_ appeared. This showed that the α-NH_2_ of WPH had been replaced by groups. More information appeared after subtracting the transmission rate of QWPH from WPH (figure 8*c*). The peak at 3493.05 cm^−1^ is the characteristic absorption peak for HR_2_C–OH, showing the epoxy group of dodecyldialkyldimethylammonium chloride had been cleaved and was added with H. The peaks at 3320.14 and 1593 cm^−1^ indicate the R_2_NH in the compound structure which means the WPH had been quaternized.

**Figure 9.**
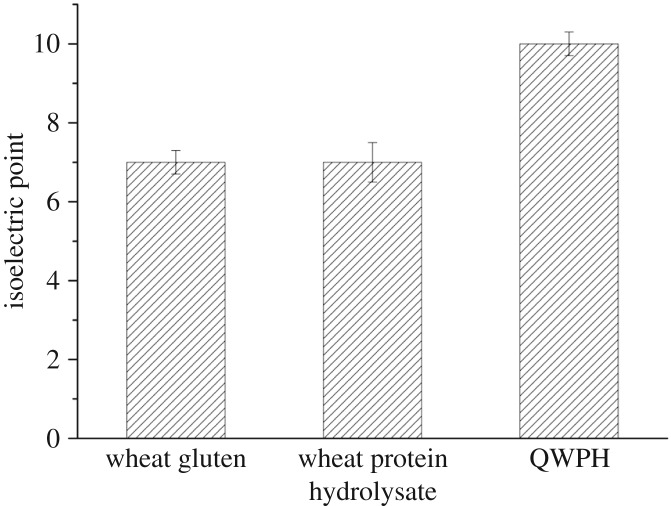
Isoelectric point of wheat gluten, WPH and QWPH.

The isoelectric point of the QWPH was raised to be 10, compared with the isoelectric point of wheat gluten and WPH (figure 9).

### 3.3. Application of quaternized wheat protein hydrolysate

Three series of conditioning shampoo were made according to the formula mentioned in Material and methods, adding different conditioners including CHEC (cationic hydroxylethylcellulose), WPH and QWPH to test the effect of wheat protein hydrolysate. Each series was designed with different conditioner content, that is, 0.5%, 1.0%, 1.5% and 2.0%. 10% SDS-(K12) solution was used to wash the hair to represent the original status of hair.

**Figure 10.**
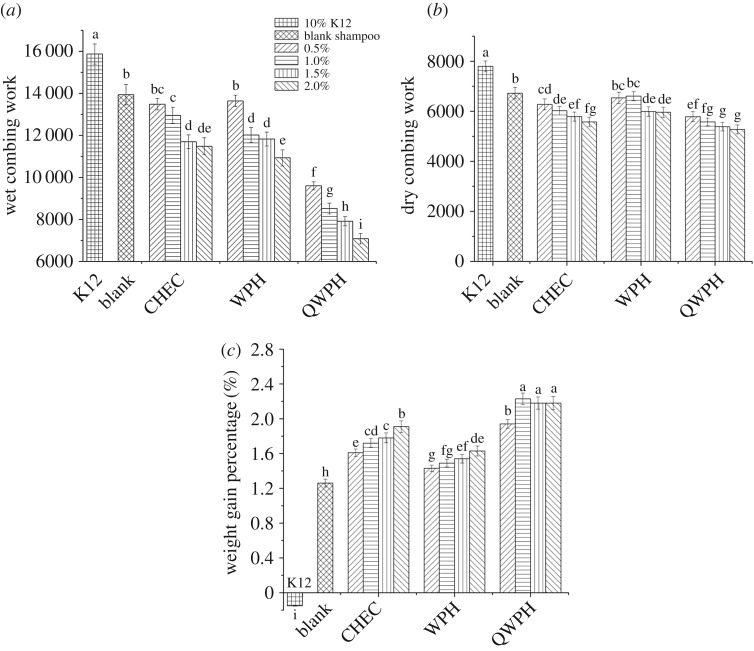
Wet combing work (*a*), dry combing work (*b*) and weight gain percentage (*c*) of hairs after treatment by different shampoos.

The friction force was monitored when the comb was passing through the hair, and the integral area of the distance–friction force curve was obtained representing the work done by the comb when it combed through the hair. Figure 10*a,b* shows similar information. For each conditioner, with the increase of the concentration, combing work became smaller and less than that of empty shampoo and that of 10% K12. Overall, the combing work showed little difference between CHEC series and WPH series at same concentration. However, the combing work significantly reduced when QWPH was added to the shampoo. This shows that the three hair conditioners were useful to improve the combing property of both wet hair and dry hair, and within the scope of the investigation, the higher the concentration was, the better the carding property was. QWPH series shampoo showed the best effect.

In figure 10*c*, the weight of the dry hair that washed by 10% K12 was slightly decreased. The weight gain rate of CHEC and WPH series increased with the increase of concentration. The highest weight gain rate of QWPH series appeared at the concentration of 2%. The weight gain rates of all three series were greater than that of blank shampoo and the order of weight gain rates was QWPH series > CHEC series > WPH series. QWPH and CHEC were both cationic, making them easy to attach to hair. Apart from cationic group, the amino acid residue of QWPH could also facilitate the attachment to hair. It is evident that QWPH is the best conditioners from the aspect of attachment.

**Figure 11.**
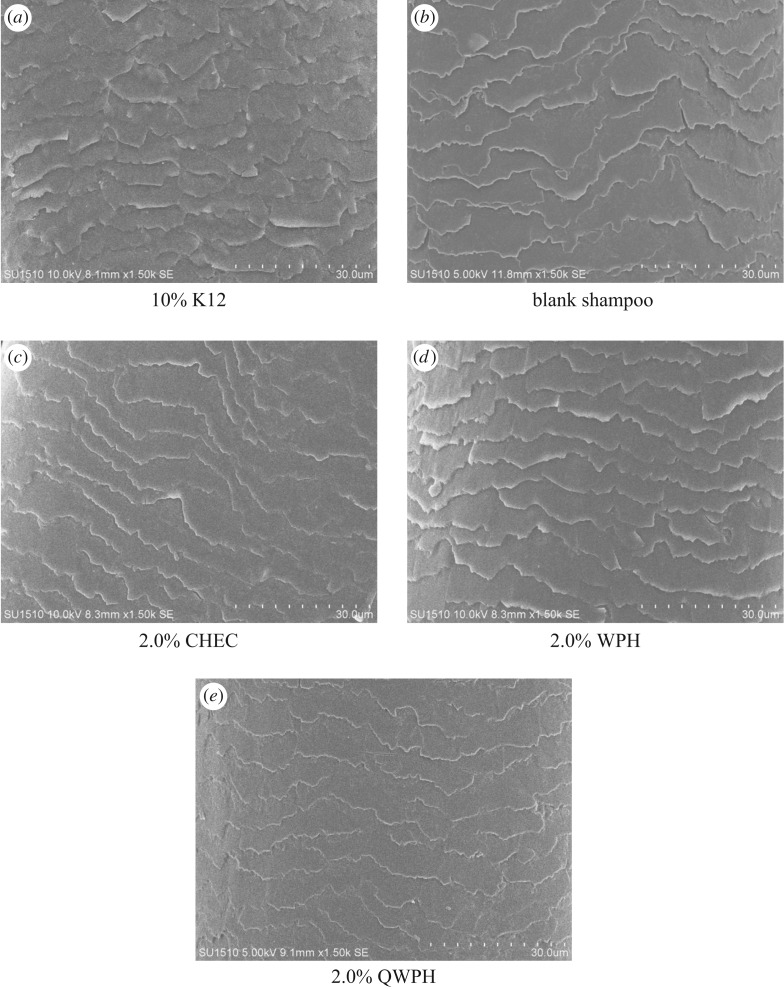
SEM images of hairs’ surface after treatment by different shampoos.

Considering combing work and weight gain rate, five kinds of shampoo were made to try out in washing hair. SEM was used to observe the surface morphology of the dry hair after washing. Morphological damage refers to a phenomenon in which the appearance and feeling of a hair deteriorates, such as peeling of the cuticle, the occurrence of wrinkling on the hair surface, or a flaw, split hair [25]. Results are shown in figure 11.

The surface morphology of the hair treated with 10% K12 in figure 11*a* which was considered to be the original status of hair indicated the hair cuticle was detached and edge warping. Figure 11*b* shows the SEM image of the blank dry hair after washing with the basic formula being a little flat but still tilted. Figure 11*c–e* shows, respectively, the images of dry hair treated with three series of shampoo, added with 2.0% of conditioner by order as CHEC, WPH, QWPH. The cuticle of hair treated with CHEC shampoo was less cocked, much smoother and partially damaged (figure 11*c*). As illustrated in figure 11*d*, the surface of the cuticle was improved to be flat but a little acute at the edge, which means WPH had some effect to recover the damaged hair. The result is in agreement with Vila's [10] result that protein hydrolysate would contribute to the sealing of the cuticles. Figure 11*e* is the SEM image of the dry hair treated with QWPH shampoo, where the surface morphology of hair is flat, smooth with a little fracture. Obviously, QWPH shampoo had a better effect than other series of shampoo, as the cationic protein hydrolysate synthesized the properties of protein hydrolysate and cationic polymer.

## 4. Conclusion

In this study, enzymatic hydrolysis and cationization with EDDAC of wheat proteins were conducted to improve properties such as solubility, emulsifying ability and disassociation behaviour to enhance the binding ability with damaged hair and the effect was obvious when used in shampoo for the recovery of damaged hair. During the enzymatic hydrolysis, the optimal conditions for enzymatic hydrolysis were determined to be pH 8.2, 55°C with Alcalase for 60 min. After hydrolysis, the DH, NSI, foaming capacity index, foam stability index, EAI and ESI of hydrolysate with 58.71% of short-chain peptides (less than 1000 Da) were 8.81%, 39.07%, 225%, 56.67%, 9.62 m^2^ g^−1^ and 49.08, respectively. After cationization the isoelectric point of WPH was raised from 7.0 to 10, which could facilitate the quaternized protein hydrolysate to adhere to the surface of hair at the range of pH 5–6 of hair care products to form more disulfide bonds. The results showed the shampoo with QWPH possessed excellent properties for recovering damaged hair, making the surface of hair smooth and compact.

## Data accessibility

The supporting data can be downloaded from the link: https://mega.nz/#!o5IzDbxR!lWIoWTELnmqeb-JUH998IPxwxkF-goyfmdvBWEkLLfQ or from the Dryad Digital Repository (https://doi.org/10.5061/dryad.c5b7t) [26]. No code is needed.
